# miR-195 Regulates Proliferation and Apoptosis through Inhibiting the mTOR/p70s6k Signaling Pathway by Targeting HMGA2 in Esophageal Carcinoma Cells

**DOI:** 10.1155/2017/8317913

**Published:** 2017-04-09

**Authors:** Yong Li, Dapeng Wu, Pei Wang, Xiaohui Li, Gongning Shi

**Affiliations:** Department of Cardiothoracic Surgery, Huaihe Hospital of Henan University, Kaifeng 475000, China

## Abstract

miR-195 is related to tumorigenesis and frequently inhibits cell proliferation and promotes apoptosis in various cancers, including esophageal carcinoma (EC). The mTOR/p70s6k signaling pathway, which is the major target pathway for HMGA2, regulates the survival and cell proliferation of many tumors and is commonly active in EC. The relationships of miR-195, HMGA2, and the mTOR/p70s6k signaling pathway in EC, however, remain unknown. In the present study, we found that the miR-195 level was significantly downregulated in EC tissues, while the mRNA expressions of HMGA2 were significantly upregulated. Dual-luciferase reporter assay demonstrated that HMGA2 is a target of miR-195. MTT assay and flow cytometry revealed that miR-195 overexpression inhibited cell proliferation and induced apoptosis by targeting HMGA2. We also found that HMGA2 restored the inhibitory effect of miR-195 on phosphorylation of mTOR and p70S6K. Furthermore, rapamycin, a specific inhibitor of the mTOR/p70S6K signaling pathway, decreased the levels of Ki-67 and Bcl-2/Bax ratio, inhibited cell proliferation, and promoted apoptosis in EC cells. In conclusion, upregulation of miR-195 significantly suppressed cell growth and induced apoptosis of EC cells via suppressing the mTOR/p70s6k signaling pathway by targeting HMGA2.

## 1. Introduction

Esophageal carcinoma (EC), one of the most common and deadly malignance worldwide, is frequently diagnosed in East Asia, especially in Northern China [1, 2]. Although treatment technology has been improved, the prognosis of patients with EC remains unfavorable [3]. Moreover, EC has been associated with a low 5-year survival rate of approximately 10% due to a frequent local invasion and distant metastasis [4]. Therefore, it is urgently required to explore the molecular mechanism of carcinogenesis and a biomarker for early diagnosis of EC, which may provide new therapeutic strategies to improve the long-term survival of patients with EC.

MicroRNAs (miRs), a class of small noncoding, single-stranded RNA with approximately 18–25 nucleotides, generally regulate the posttranscriptional level of target messenger RNA (mRNA) expression by binding to the complementary domains of the 3′-untranslated region (UTR) of their target mRNA [5, 6]. It is reported that miRs regulate the posttranscriptional levels of more than 30% human genes [7]. miRs are closely related to diverse biological functions and pathologic processes, including cell proliferation, differentiation, apoptosis, and self-renewal [8]. Studies have shown that miRs can act as oncogenes or tumor suppressors by targeting cancer-related genes in the development and carcinogenesis of EC and might be used as a diagnostic biomarker for human cancers [9–11]. For example, miR-337 was oncogenic and upregulated in EC cells and could markedly promote proliferation and inhibit apoptosis by suppressing PTEN expression [12]. miR-625 was reported to exhibit downregulation in EC and increase cell proliferation and invasion by targeting Sox2 [13]. miR-195 was demonstrated to inhibit cell proliferation and promote apoptosis in human cervical cancer and to suppress tumor growth and angiogenesis in breast cancer by different function routes [14, 15]. A previous study has indicated that miR-195 was downregulated in esophageal squamous cell carcinoma and inhibited cell proliferation and invasion by targeting Cdc42 [16]. However, the underlying molecular mechanism of miR-195 in EC remains largely unknown.

mTOR, the mammalian target of rapamycin, belongs to phosphoinositide 3-kinase- (PI3K-) related kinase family and is evolutionarily conserved [17, 18]. The mTOR/p70s6k signaling pathway regulates the survival and cell proliferation of many tumors and is commonly active in various cancers including EC [19, 20]. Therefore, mTOR has been regarded as a crucial target for the treatment of human cancer. The p70s6 kinase (p70s6k) is a major effector of mTOR in the downstream of the mTOR/p70s6k signaling pathway, which is a major effector in the downstream of the PI3K/AKT signaling pathway [21]. Research has indicated that the mTOR/p70s6k signaling pathway was activated in esophageal squamous cell carcinoma and rapamycin, suppressed mTOR expression and phosphorylation of p70s6k, arrested cell cycle at the G0/G1 phase, and induced apoptosis of EC cells [22].

High-mobility group protein A2 (HMGA2), a major nonhistone chromosomal protein and DNA-binding transcription factor, interacts with many different transcription factors, regulates the expression patterns of genes, and functions as a key regulator of foundational cellular processes, such as cell cycle, transformation, proliferation, and apoptosis [23–25]. HMGA2 induces oncogenesis and is upregulated in various tumors [26, 27]. The PI3K/AKT/mTOR/p70s6k signaling pathway is the major target pathway associated with HMGA2 expression, and HMGA2 can activate the PI3K/AKT/mTOR/p70s6k signaling pathway directly by inducing phosphorylation of AKT, mTOR, and p70s6k in human umbilical cord blood-derived stromal cells (hUCBSCs) [28]. However, the relationship between the mTOR/p70s6k signaling pathway and HMGA2 in EC is still unknown. In addition, our bioinformatics analysis predicted that HMGA2 is a putative target of miR-195. But no studies have been reported about the relationship between miR-195 and HMGA2 in EC.

In our study, we verified the expression of miR-195 and HMGA2 in EC tissues and adjacent normal epithelium tissues. Then, we studied the relationship between miR-195 and HMGA2. Finally, we investigated the role of miR-195, HMGA2, and the mTOR/p70S6K signaling pathway in the regulation of cell proliferation and apoptosis in EC.

## 2. Materials and Methods

### 2.1. Clinical Specimens and Cell Lines

Fifteen surgically resected EC tissues and adjacent normal epithelium tissues were obtained from Huaihe Hospital of Henan University. The EC tissues and adjacent normal epithelium tissues were immediately collected after surgical resection, immersed with RNAlater reagent (TaKaRa, Dalian, China) for 30 min and subsequently frozen at −80°C until use. The primary tumor tissues were confirmed by pathological analysis performed by a pathologist and classified according to the World Health Organization classification. The tumor cellularity of the selected EC samples was more than 70%. None of the patients with EC had received radiotherapy, chemotherapy, or other esophageal surgeries before esophagectomy. The study was approved by the Local Research Ethics Committee of Huaihe Hospital of Henan University. Written informed consent was obtained from all patients or their guardians. Human EC cell lines (EC109 and EC9706) were obtained from the Chinese Science Institute (Shanghai, China). All cells were maintained in Dulbecco's modified Eagle's medium (DMEM; Genom, Hangzhou, China) supplemented with 10% fetal bovine serum (FBS; Hyclone, Logan, UT, USA), 100 U/ml of penicillin, and 100 *μ*g/ml of streptomycin (Gibco, Gaithersburg, MD, USA) at 37°C with 5% CO_2_.

### 2.2. Cell Transfection

EC109 and EC9706 cells were seeded in 6-well plates and transfected with miR-195 mimics, miR-control, anti-miR-195, anti-miR-control, pcDNA-HMGA2, pcDNA empty vector, si-HMGA2, or si-control (GenePharma, Shanghai, China) by Lipofectamine™ 2000 (Invitrogen, Carlsbad, CA, USA) following the manufacturer's protocol when the cells were grown to 80% confluency.

### 2.3. Quantitative Real-Time PCR

Total RNA was isolated from fresh frozen samples of EC patients and cell lines using the Trizol Reagent (Invitrogen). cDNAs were synthesized from 500 ng of total RNA by using PrimeScript™ one-step RT-PCR kit (TaKaRa). The expression of miR-195 and HMGA2 was determined by real-time PCR reaction which was executed with SYBR Premix Ex Taq II (TaKaRa) on a CFX 96 real-time PCR thermocycler (TaKaRa). U6 small nuclear RNA (snRNA) expression was used as the internal control.

### 2.4. Dual-Luciferase Assay

The 3′-UTR of HMGA2 containing putative miR-195 binding sites (HMGA2-3′-UTR-wt) predicted by TargetScan (http://www.targetscan.org/) and a mutant miR-195 binding site (HMGA2-3′-UTR-mut) was synthesized and cloned into the pMIR-REPORT Luciferase Expression Reporter Vector (Invitrogen). For reporter assays, 50 pmol miR-195 or miR-control was cotransfected with 50 ng of pMIR-REPORT HMGA2-3′-UTR-wt or pMIR-REPORT HMGA2-3′-UTR-mut into EC109 and EC9706 cells placed in 24-well plates using Lipofectamine 2000 (Invitrogen). At 24 h after transfection, the Dual-Luciferase Reporter Assay System (Promega, Madison, WI, USA) was used to analyze luciferase activities and Renilla activity was used as the internal control.

### 2.5. Western Blot Analysis

EC109 and EC9706 cells were collected and total cell lysate was obtained by using RIPA buffer (ZhongShan JinQiao, Beijing, China). BCA protein assay kits (CoWin Biotechnology, Beijing, China) were used to detect the protein concentrations. Equal amounts of protein extracts from different groups were subjected to 12% SDS-PAGE and transferred onto nitrocellulose membranes (Millipore, Boston, MA, USA). After blocking in 5% nonfat milk for 1 h, the membranes were probed with indicated primary antibodies against HMGA2, t-p70s6k, p-p70s6k, t-mTOR, p-mTOR, Ki-67, Bcl-2, Bax, and *β*-actin (Santa Cruz Biotechnology, Santa Cruz, CA, USA) and incubated overnight at 4°C. After washing with TBS three times, the immunoblotted membranes were further incubated with corresponding horseradish peroxidase- (HRP-) conjugated secondary antibody (Santa Cruz Biotechnology) for 2 h. Protein bands were finally visualized using chemiluminescence (Millipore, MA, USA) and normalized to *β*-actin.

### 2.6. Cell Proliferation Assay

EC109 and EC9706 cells were seeded into 96-well plates (BD Biosciences, San Jose, CA, USA) and were incubated in an incubator of 5% CO_2_ at 37°C. At 24 h or 48 h after transfection with miR-195 mimics, miR-control, pcDNA, or pcDNA-HMGA2, cells were incubated with 3-(4,5)-dimethylthiahiazo(-z-y1)-3,5-di-phenytetrazoliumromide (MTT) (20 *μ*l of 5 mg/ml) (KeyGen BioTech, Nanjing, China) reagent for 4 h at 37°C. Following incubation, the supernatants were then discarded and 150 *μ*l of dimethyl sulfoxide (Invitrogen) was used to solubilize the purple precipitates of formazan. The absorbances of each well at 490 nm were detected using an automatic micro plate reader (Gene, HK). The experiments were repeated in triplicates.

### 2.7. Flow Cytometric Detection of Apoptosis

EC109 and EC9706 cells were seeded into 96-well plates (BD Biosciences, San Jose, CA, USA) and were incubated in an incubator of 5% CO_2_ at 37°C. At 48 h after transfection with miR-195 mimics, miR-control, pcDNA, or pcDNA-HMGA2, cells were collected and stained with Annexin V-FITC/PI (BestBio Biotechnology Co., Ltd., Shanghai, China) according to the manufacturer's instructions. Finally, the apoptosis of cells were determined using flow cytometer (Becton Dickinson, San Jose, CA, USA).

### 2.8. Statistical Analysis

All data were analyzed by SPSS 13.0 software. Values were expressed as means ± SEM or mean ± SD. Statistical comparisons were calculated with Student's *t*-test or one-way analysis of variance (ANOVA). *P* values less than 0.05 were considered to be statistically significant.

## 3. Results

### 3.1. miR-195 and HMGA2 Expressions in Human EC Tissues

**Figure 1:**
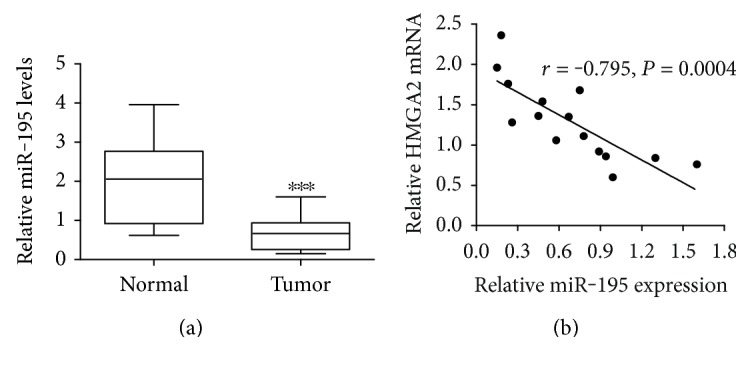
miR-195 is downregulated and HMGA2 is upregulated in human EC tissues. (a) The expression of *miR-195* in fifteen surgically resected EC tissues and adjacent normal epithelium tissues was examined. (b) Correlation between *miR-195* and *HMGA2* mRNA expression in EC tissues. *miR-195* and *HMGA2* mRNA expression profiles were determined by qRT-PCR. The relative expression of *miR-195* and *HMGA2* mRNA was normalized to the U6 snRNA. Values are presented as the mean ± SD, *n* = 3. ^∗∗∗^*P* < 0.001.

qRT-PCR was used to analyze the expression levels of *miR-195* and *HMGA2* mRNA in 15 EC tissues. As shown in Figure 1(a), *miR-195* expression was lower in EC tissues as compared with that in adjacent normal epithelium tissues.  Figure 1(b) showed a statistically significant negative correlation between *HMGA2* mRNA and *miR-195* expressions in 15 EC tissues.

### 3.2. miR-195 Directly Regulates HMGA2 Expression by Binding to the 3′-UTR of HMGA2 in EC Cell Lines

**Figure 2:**
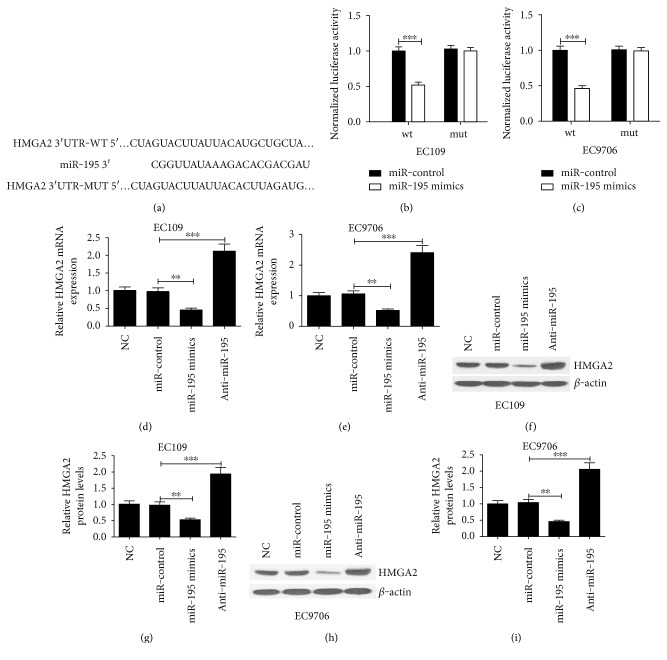
miR-195 directly targets the 3′-UTR of HMGA2 to regulate HMGA2 expression in EC cell lines. (a) Prediction of the HMGA2 mRNA 3′-UTR sites targeted by miR-195. The underline showed the mutated nucleotides on the 3′-UTR of HMGA2. Relative luciferase activity in (b) EC109 and (c) EC9706 cells. Cells were transfected with pMIR-REPORT vector containing HMGA2-3′-UTR-wt or HMGA2-3′-UTR-mut together with miR-195 mimics or miR-control for 24 h. Renilla activity was used as the internal control. The expressions of *HMGA2* mRNA in (d) EC109 and (e) EC9706 cells transfected with miR-195 mimics, anti-miR-195, or miR-control were detected by qRT-PCR. The expressions of HMGA2 protein in (f and g) EC109 and (h and i) EC9706 cells transfected with miR-195 mimics, anti-miR-195, or miR-control were detected by western blot. The expressions of HMGA2 mRNA and protein were normalized to U6 snRNA and *β*-actin. Values are presented as the mean ± SD, *n* = 3. ^∗∗^*P* < 0.01, ^∗∗∗^*P* < 0.001.

The predicted HMGA2 3′-UTR binding site for miR-195 is shown in Figure 2(a) based on the TargetScan software and bioinformatics analysis. To identify whether miR-195 could directly target the HMGA2 gene, we constructed luciferase reporter vectors pMIR-REPORT HMGA2-3′-UTR-wt and pMIR-REPORT HMGA2-3′-UTR-mut containing the luciferase coding sequence in the downstream of the 3′-UTR of HMGA2. The luciferase reporter system showed that miR-195 decreased approximately half of the luciferase activity of pMIR-REPORT HMGA2-3′-UTR-wt compared with that of the miR-control groups, while no obvious inhibitory effect on luciferase activity was observed in that of pMIR-REPORT HMGA2-3′-UTR-mut for EC109 and EC9706 cells (Figures 2(b) and 2(c)). The results indicated that HMGA2 was a target of miR-195. Furthermore, to confirm whether miR-195 downregulated HMGA2 expression, EC109 and EC9706 cells were transfected with miR-195 mimics, anti-miR-195, or miR-control. As shown in Figures 2(d) and 2(e), the expression of *HMGA2* mRNA was significantly reduced by miR-195 overexpression but strikingly improved by miR-195 downregulation in EC109 and EC9706 cells. Besides, the HMGA2 protein expression was significantly decreased by miR-195 overexpression but significantly increased by miR-195 downregulation in both EC109 (Figures 2(f) and 2(g)) and EC9706 cells (Figures 2(h) and 2(i)). These data suggested that miR-195 could specifically bind to the 3′-UTR of HMGA2 and negatively regulate HMGA2 expression.

### 3.3. miR-195 Overexpression Inhibits Cell Proliferation and Induces Apoptosis in EC Cell Lines by Targeting HMGA2

**Figure 3:**
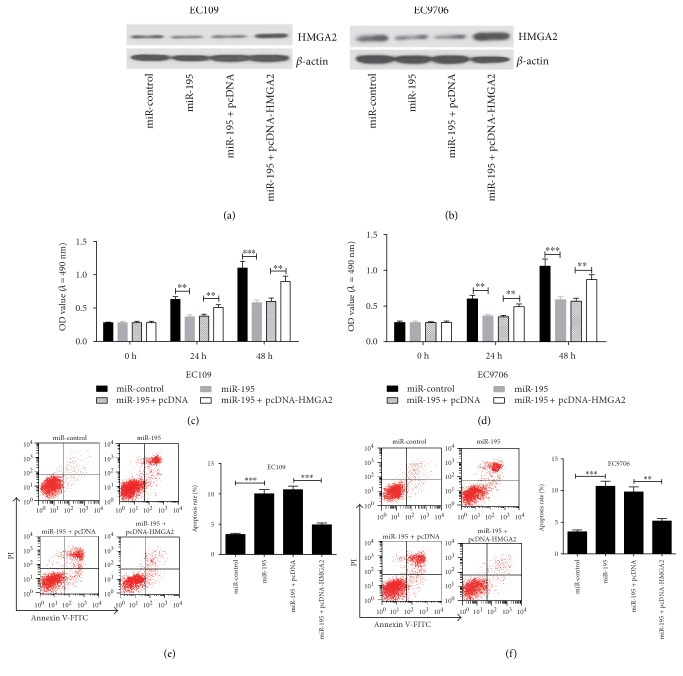
Overexpression of HMGA2 relieved the effect of miR-195 on proliferation and apoptosis in EC cell lines. EC109 and EC9706 cells were transfected with miR-control or miR-195 mimics or cotransfected miR-195 mimics with pcDNA or pcDNA-HMGA2. The protein expressions of HMGA2 in EC109 (a) and EC9706 (b) cells were detected by western blot. Cell proliferation in EC109 (c) and EC9706 (d) cells was assessed by MTT assay at 24 and 48 h. Cell apoptosis in EC109 (e) and EC9706 (f) cells was detected by flow cytometry via double staining of cells with Annexin V-FITC and propidium iodide (PI) at 48 h. Values are presented as the mean ± SD, *n* = 3. ^∗∗^*P* < 0.01, ^∗∗∗^*P* < 0.001.

In order to explore the role of miR-195 overexpression on the viability and apoptosis of EC cell lines, EC109 and EC9706 cells were transfected with miR-control or miR-195 mimics or cotransfected miR-195 mimics with pcDNA or pcDNA-HMGA2. As expected, pcDNA-HMGA2 dramatically reduced the inhibition effect of miR-195 mimics on the expression of HMGA2 in EC109 (Figure 3(a)) and EC9706 cells (Figure 3(b)). After 24 h or 48 h of transfection, cell proliferation was detected by MTT assay. The results indicated that the miR-195 mimic significantly inhibited cell proliferation in both EC109 and EC9706 cells at 24 h and 48 h, whereas the cell proliferation inhibition by miR-195 mimic was alleviated due to the transfection of pcDNA-HMGA2 (Figures 3(c) and 3(d)). Then, we investigated the apoptosis of EC109 and EC9706 cells by flow cytometry. The results showed that miR-195 mimics significantly increased apoptosis in both EC109 and EC9706 cells, while the miR-195-induced apoptosis rate was reverted as a result of HMGA2 overexpression (Figures 3(e) and 3(f)).

### 3.4. HMGA2 Overexpression Recuperated the mTOR/p70S6K Signaling Pathway Suppressed by miR-195

**Figure 4:**
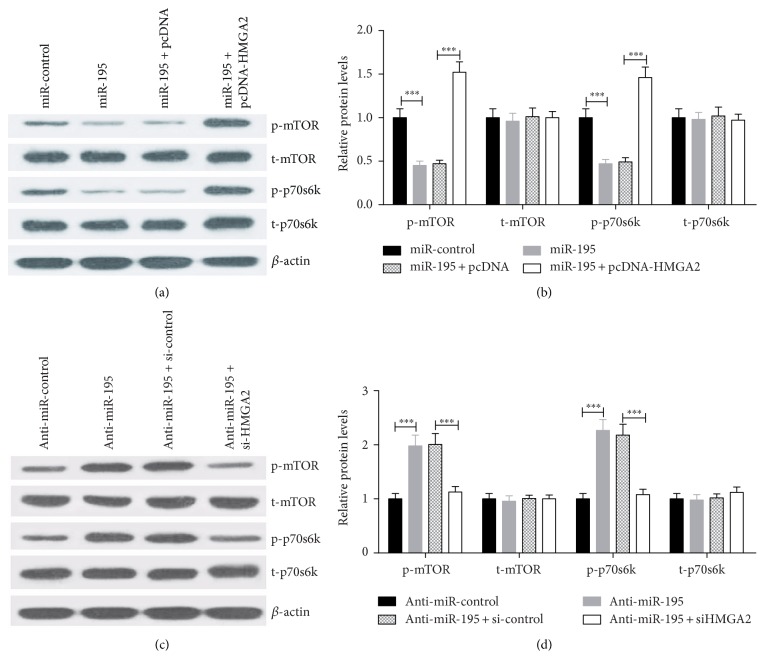
miR-195 suppressed the mTOR/p70s6k signaling pathway by inhibiting HMGA2 expression in EC9706 cells. (a and b) Western blot analysis for relative proteins p-mTOR, t-mTOR, p-p70s6k, and t-p70s6k in EC9706 cells transfected with miR-control or miR-195 mimics or cotransfected miR-195 mimics together with pcDNA or pcDNA-HMGA2. (c and d) Western blot analysis for relative proteins p-mTOR, t-mTOR, p-p70s6k, and t-p70s6k in EC9706 cells transfected with anti-miR-control or anti-miR-195 or cotransfected anti-miR-195 together with si-control or si-HMGA2. *β*-actin was used as an internal control. Values are presented as the mean ± SD, *n* = 3. ^∗∗∗^*P* < 0.001.

Previous studies have indicated that the mTOR/p70S6K signaling pathway, a major target pathway associated with HMGA2, can be activated by HMGA2 via inducing the phosphorylation of mTOR and p70S6K in human umbilical cord blood-derived stromal cells (hUCBSCs) [28]. Since miR-195 directly targets HMGA2 and regulates the expression of HMGA2, we hypothesize that miR-195 regulates the mTOR/p70s6k signaling pathway by inhibiting HMGA2 expression. EC9706 cells were transfected with miR-195 mimics or miR-195 mimics+pcDNA-HMGA2 or anti-miR-195 or anti-miR-195+si-HMGA2 to investigate the effect of miR-195 on mTOR and p70s6k. As presented in Figures 4(a) and 4(b), western blot showed that miR-195 overexpression obviously suppressed the phosphorylation of mTOR and p70s6k, which was alleviated by HMGA2 overexpression. In addition, downregulation of miR-195 dramatically promoted the phosphorylation of mTOR and p70s6k, which was inhibited by HMGA2 silencing (Figures 4(c) and 4(d)). These data suggested that HMGA2 overexpression strikingly relieved the inhibition effect of miR-195 on the mTOR/p70S6K signaling pathway.

### 3.5. Inhibition of the mTOR/p70S6K Signaling Pathway Suppresses Cell Proliferation and Promotes Apoptosis in EC9706 Cells

**Figure 5:**
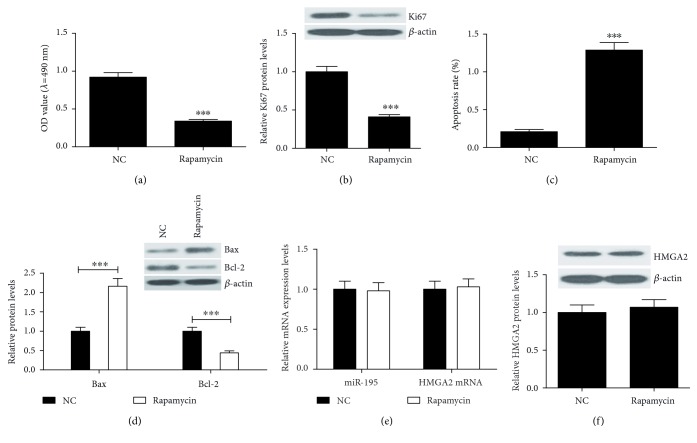
Rapamycin inhibits the cell proliferation and induced apoptosis via suppression of the mTOR/p70S6K signaling pathway in EC9706 cells. EC9706 cells were treated with 100 nmol/l rapamycin for 24 h. (a) Cell viability was examined by MTT assay. (b) Western blot analysis of Ki-67 expression. (c) Apoptosis rates in EC9706 cells were assessed by flow cytometry. (d) Western blot analysis of Bcl-2 and Bax expression. (e) qRT-PCR was used to determine the expressions of miR-195 and HMGA2 mRNA. (f) Western blot analysis of HMGA2 protein expression. *β*-actin was used as an internal control. Values are presented as the mean ± SD, *n* = 3. ^∗∗^*P* < 0.01, ^∗∗∗^*P* < 0.001.

To investigate the role of the mTOR/p70S6K signaling pathway in cell proliferation and apoptosis, EC9706 cells were treated with 100 nmol/l rapamycin, a specific inhibitor of the mTOR/p70S6K signaling pathway, for 24 h. MTT assay, flow cytometry, and western blot were used to evaluate cell proliferation, apoptosis, and the related protein of EC9706 cells, respectively. EC9706 cells treated with rapamycin showed a significant decrease of cell viability (Figure 5(a)) and Ki-67 expression (Figure 5(b)) compared with those of the NC group. Moreover, rapamycin significantly increased apoptosis rates (Figure 5(c)) and Bax/Bcl-2 ratio (Figure 5(d)) of EC9706 cells. Besides, rapamycin was found to have no obvious effects on miR-195 and HMGA2 expression (Figures 5(e) and 5(f)). All the data indicated that the mTOR/p70s6k signaling pathway may be involved in the proliferation and apoptosis modulated by miR-195 in EC9706 cells.

## 4. Discussion

It is reported that more than 30% of human gene expression are related to miRs [6]. Numerous studies have suggested that miRs can regulate carcinogenesis and progression of malignant cancers by target binding to the 3′-UTR of mRNAs [29]. It has been found that miR-195 has a crucial role in the progression of different cancers. For instance, miR-195 expression was significantly decreased in cervical cancer cell lines and overexpression of miR-195 exerted a significant inhibitory impact on the proliferation of cervical cancer cells and induced apoptosis in vitro by targeting cyclin D1 [16]. miR-195 inhibited the tumor angiogenesis and growth by suppressing IRS1 in breast cancer [17]. In prostate cancer cells, miR-195 functions as a tumor suppressor gene by suppressing HMGA1 [30]. In esophageal squamous cell carcinoma cells, Fu et al. manifested that miR-195 downregulation inhibited cell proliferation and invasion by targeting Cdc42 [16]. Consistent with the above results, we found that miR-195 was significantly low expressed in EC tissues and upregulation of miR-195 significantly inhibited the cell proliferation and induced apoptosis. These results strongly indicated the potential tumor-suppressive role of miR-195 in EC.

HMGA2 was found upregulated in various cancers, such as lung cancer [31], breast cancer [32], and oral squamous cell carcinomas [33]. Liu et al. reported that HMGA2, a target of miR let-7, was upregulated in EC cells and was involved in EC carcinogenesis [34]. However, the relationship between miR-195 and HMGA2 in EC has not been reported. In our study, dual-luciferase assay demonstrated that there existed a significant decrease of the relative luciferase activity for the reporter vector pMIR-REPORT containing wild-type 3′-UTR of HMGA2 but no obvious change in the mutant. The level of HMGA2 protein was inversely correlated with miR-195 expression. Moreover, cell proliferation inhibition and apoptosis of EC cells induced by overexpression of miR-195 were alleviated by overexpression of HMGA2. These findings indicated that HMGA2 was a direct target of miR-195, and miR-195 played the potential tumor-suppressive role in EC cells via targeting HMGA2.

The mTOR/p70s6k signaling pathway is implicated in cell proliferation, invasion, and metastasis in many cancers including squamous cell carcinomas of the head and neck [35], hepatocellular carcinomas [36], breast cancer [37], and EC [38]. Jia et al. showed that miR-223 restrained the cell proliferation via the mTOR/p70s6k signaling pathway by targeting IGF-1R in HeLa cells [39]. The present study revealed that overexpression of miR-195 suppressed the key components mTOR and p70S6K phosphorylation. HMGA2 overexpression rescued the inhibition of mTOR and p70S6K phosphorylation by miR-195 overexpression in EC9706 cells, whereas HMGA2 downregulation inhibited the promotion of mTOR and p70S6K phosphorylation by miR-195 downregulation. These results manifested that miR-195 inhibited the mTOR/p70s6k signaling pathway by targeting HMGA2.

Previous reports have demonstrated that rapamycin can inhibit the mTOR/p70s6k signaling pathway via inhibiting mTOR and p70S6K phosphorylation [40]. Our study verified the role of the mTOR/p70s6k signaling pathway on the proliferation and apoptosis of EC cells. Rapamycin significantly inhibited cell proliferation and promoted cell apoptosis. Besides, the expressions of Ki-67 and Bcl-2/Bax ratio were also decreased in rapamycin-treated cells. However, rapamycin had no effects on miR-195 and HMGA2 expressions. These findings indicated that inhibition of the mTOR/p70s6k signaling pathway by rapamycin led to cell proliferation inhibition and apoptosis promotion of EC cells.

## 5. Conclusion

In summary, our findings demonstrated that miR-195 expression was downregulated in EC cells and inversely correlated with HMGA2 levels. Overexpression of miR-195 evidently restrained proliferation and promoted apoptosis of EC cells through the mTOR/p70s6k signaling pathway by targeting HMGA2. Therefore, miR-195 may function as a tumor suppressor and be developed as the potential diagnostic and therapeutic target for EC.

## References

[1] Parkin D. M., Bray F., Ferlay J., Pisani P. (2001). Estimating the world cancer burden: Globocan 2000. *International Journal of Cancer*.

[2] Jemal A., Siegel R., Xu J., Ward E. (2010). Cancer statistics, 2010. *CA: A Cancer Journal for Clinicians*.

[3] Montesano R., Holestein M., Hainaui P. (1996). Genetic alterations in esophageal cancer and their relevance to etiology and pathogenesis: a review. *International Journal of Cancer*.

[4] Kim T., Grobmyer S. R., Smith R. (2011). Esophageal cancer—the five year survivors. *Journal of Surgical Oncology*.

[5] Ryan B. M., Robles A. I., Harris C. C. (2010). Genetic variation in microRNA networks: the implications for cancer research. *Nature Reviews Cancer*.

[6] Bartel D. P. (2004). MicroRNAs: genomics, biogenesis, mechanism, and function. *Cell*.

[7] Giordano S., Columbano A. (2013). MicroRNAs: new tools for diagnosis, prognosis, and therapy in hepatocellular carcinoma?. *Hepatology*.

[8] Schmittgen T. D. (2008). Regulation of microRNA processing in development, differentiation and cancer. *Journal of Cellular and Molecular Medicine*.

[9] Liu M., Lang N., Qiu M. (2011). miR-137 targets Cdc42 expression, induces cell cycle G1 arrest and inhibits invasion in colorectal cancer cells. *International Journal of Cancer*.

[10] Song Y. X., Yue Z. Y., Wang Z. N. (2011). MicroRNA-148b is frequently down-regulated in gastric cancer and acts as a tumor suppressor by inhibiting cell proliferation. *Molecular Cancer*.

[11] Zhang X., Nie Y., Du Y., Cao J., Shen B., Li Y. (2012). MicroRNA-181a promotes gastric cancer by negatively regulating tumor suppressor KLF6. *Tumor Biology*.

[12] Cai Y., He T., Liang L., Zhang X., Yuan H. (2016). Upregulation of microRNA-337 promotes the proliferation of endometrial carcinoma cells via targeting PTEN. *Molecular Medicine Reports*.

[13] Wang Z., Qiao Q., Chen M. (2014). miR-625 down-regulation promotes proliferation and invasion in esophageal cancer by targeting Sox2. *FEBS Letters*.

[14] Li Z., Wang H., Wang Z., Cai H. (2015). miR-195 inhibits the proliferation of human cervical cancer cells by directly targeting cyclin D1. *Tumor Biology*.

[15] Wang Y., Zhang X., Zou C. (2016). miR-195 inhibits tumor growth and angiogenesis through modulating IRS1 in breast cancer. *Biomedicine & Pharmacotherapy*.

[16] Fu M. G., Li S., Yu T. T. (2013). Differential expression of miR-195 in esophageal squamous cell carcinoma and miR-195 expression inhibits tumor cell proliferation and invasion by targeting of Cdc42. *FEBS Letters*.

[17] Hay N., Sonenberg N. (2004). Upstream and downstream of mTOR. *Genes & Development*.

[18] Han S., Khuri F. R., Roman J. (2006). Fibronectin stimulates non–small cell lung carcinoma cell growth through activation of Akt/mammalian target of rapamycin/S6 kinase and inactivation of LKB1/AMP-activated protein kinase signal pathways. *Cancer Research*.

[19] Martelli A. M., Evangelisti C., Follo M. Y. (2011). Targeting the phosphatidylinositol 3-kinase/Akt/mammalian target of rapamycin signaling network in cancer stem cells. *Current Medicinal Chemistry*.

[20] Hou G., Xue L., Lu Z., Fan T., Tian F., Xue Y. (2007). An activated mTOR/p70S6K signaling pathway in esophageal squamous cell carcinoma cell lines and inhibition of the pathway by rapamycin and siRNA against mTOR. *Cancer Letters*.

[21] Heinonen H., Nieminen A., Saarela M. (2008). Deciphering downstream gene targets of PI3K/mTOR/p70S6K pathway in breast cancer. *BMC Genomics*.

[22] Hou G., Xu P., Xue Y., Xue L. (2007). mTOR/p70S6K signaling pathway constitutively activated in esophageal squamous cell carcinoma cell lines and inhibition of the pathway by rapamycin and siRNA against mTOR. *Cancer Research*.

[23] Sgarra R., Rustighi A., Tessari M. A. (2004). Nuclear phosphoproteins HMGA and their relationship with chromatin structure and cancer. *FEBS Letters*.

[24] Young A. R., Narita M. (2007). Oncogenic HMGA2: short or small?. *Genes & Development*.

[25] Rahman M. M., Qian Z. R., Wang E. L. (2009). Frequent overexpression of HMGA1 and 2 in gastroenteropancreatic neuroendocrine tumours and its relationship to let-7 downregulation. *British Journal of Cancer*.

[26] Narita M., Narita M., Krizhanovsky V. (2006). A novel role for high-mobility group A proteins in cellular senescence and heterochromatin formation. *Cell*.

[27] Fedele M., Palmieri D., Fusco A. (2010). HMGA2: a pituitary tumour subtype-specific oncogene?. *Molecular and Cellular Endocrinology*.

[28] Yu K. R., Park S. B., Jung J. W. (2013). HMGA2 regulates the in vitro aging and proliferation of human umbilical cord blood-derived stromal cells through the mTOR/p70S6K signaling pathway. *Stem Cell Research*.

[29] Clop A., Marcq F., Takeda H. (2006). A mutation creating a potential illegitimate microRNA target site in the myostatin gene affects muscularity in sheep. *Nature Genetics*.

[30] Zhang X., Tao T., Liu C. (2016). Downregulation of miR-195 promotes prostate cancer progression by targeting HMGA1. *Oncology Reports*.

[31] Sarhadi V. K., Wikman H., Salmenkivi K. (2006). Increased expression of high mobility group A proteins in lung cancer. *The Journal of Pathology*.

[32] Rogalla P., Drechsler K., Kazmierczak B., Rippe V., Bonk U., Bullerdiek J. (1997). Expression of HMGI-C, a member of the high mobility group protein family, in a subset of breast cancers: relationship to histologic grade. *Molecular Carcinogenesis*.

[33] Miyazawa J., Mitoro A., Kawashiri S., Chada K. K., Imai K. (2004). Expression of mesenchyme-specific gene HMGA2 in squamous cell carcinomas of the oral cavity. *Cancer Research*.

[34] Liu Q., Lv G. D., Qin X. (2012). Role of microRNA let-7 and effect to HMGA2 in esophageal squamous cell carcinoma. *Molecular Biology Reports*.

[35] Amornphimoltham P., Patel V., Sodhi A. (2005). Mammalian target of rapamycin, a molecular target in squamous cell carcinomas of the head and neck. *Cancer Research*.

[36] Sahin F., Kannangai R., Adegbola O., Wang J., Su G., Torbenson M. (2004). mTOR and P70 S6 kinase expression in primary liver neoplasms. *Clinical Cancer Research*.

[37] van der Hage J. A., van den Broek L. J., Legrand C. (2004). Overexpression of P70 S6 kinase protein is associated with increased risk of locoregional recurrence in node-negative premenopausal early breast cancer patients. *British Journal of Cancer*.

[38] deGraffenried L. A., Friedrichs W. E., Russell D. H. (2004). Inhibition of mTOR activity restores tamoxifen response in breast cancer cells with aberrant Akt activity. *Clinical Cancer Research*.

[39] Jia C. Y., Li H. H., Zhu X. C. (2011). MiR-223 suppresses cell proliferation by targeting IGF-1R. *PloS One*.

[40] Hu L., Hofmann J., Lu Y., Mills G. B., Jaffe R. B. (2002). Inhibition of phosphatidylinositol 3′-kinase increases efficacy of paclitaxel in in vitro and in vivo ovarian cancer models. *Cancer Research*.

